# Primary Varicella Infection in a Young Adult from the Democratic Republic of the Congo: A Case Report and Mini-Review

**DOI:** 10.3390/idr16040048

**Published:** 2024-07-19

**Authors:** Andrew McNaughton, Nessika Karsenti, Jason Kwan, Asal Adawi, Saniya Mansuri, Andrea K. Boggild

**Affiliations:** 1 Department of Medicine, University of Ottawa, Ottawa, ON K1H 8M5, Canada; 2 Department of Medicine, Memorial University, Saint John’s, NF A1B 3V6, Canada; 3 Department of Medicine, University of British Columbia, Vancouver, BC V1Y 1T3, Canada; 4 Tropical Disease Unit, Toronto General Hospital, Toronto, ON M5G 2C4, Canada; 5 TMC Innovation, Texas Medical Center, Houston, TX 77030, USA; 6 Department of Medicine, University of Toronto, Toronto, ON M5S 1A1, Canada; 7 Institute of Medical Science, University of Toronto, Toronto, ON M5S 1A8, Canada

**Keywords:** chicken pox, fever in the returned traveler, herpesviridae, varicella zoster virus, viral exanthem

## Abstract

We describe a case of an immunocompetent adult male patient originally from the Democratic Republic of Congo (DRC), who was referred to our unit for a several-day history of fever and a pruritic, vesicular rash. There was initial concern in the Emergency Department for Mpox (formerly known as “monkeypox”) given the current epidemiology versus other viral etiologies. Primary varicella zoster virus (pVZV) infection was ultimately diagnosed by PCR from a swabbed, unroofed lesion, and he recovered completely with supportive management and without antiviral therapy. We herein describe how common viral exanthems may best be differentiated in an emergency or outpatient setting.

## 1. Introduction

Primary varicella is an acute, contagious, and common childhood disease caused by the varicella-zoster virus (VZV) [1]. While often benign and self-limiting, infection with VZV can be severe in infants, adults, and immunocompromised individuals [1]. Two varicella vaccines were approved in December 2002 for use in children in Canada, which have significantly reduced the burden of this disease nationally [2,3]. Much of our current understanding of VZV and its epidemiology is based on data from temperate, high-income countries. Research from countries in the tropics demonstrates that primary infection of varicella often occurs later in life when the disease is more likely to be severe [1]. Acute varicella infection presents with a pruritic, vesicular rash and fever [1]. Distinguishing varicella from other viral infections can be difficult clinically and has increasing public health implications due to the 2022 Mpox outbreak [4], along with global increases in similarly presenting vaccine-preventable viral exanthems such as measles, as well as other emerging and surging pathogens such as group A *Streptococcus* (*S. pyogenes*) and dengue.

Treatment of VZV is often supportive for children with primary varicella; however, antiviral therapy may be required in older adolescents, adults, and immunocompromised individuals to prevent severe complications including secondary bacterial skin and soft tissue infections, neurologic complications (e.g., transverse myelitis and encephalitis), hepatitis, and pneumonia [5], which can carry mortality rates of up to 30% [6]. Furthermore, multiple studies have demonstrated that the benefit of antiviral therapy in preventing secondary complications is greatest when treatment is initiated within 24 h of symptom onset [7,8]; therefore, prompt identification and treatment can improve clinical outcomes. We herein report a case of primary varicella infection in a 27-year-old male from the Democratic Republic of Congo. This case illustrates the challenge of diagnosing primary varicella infection in adults, as well as differences in epidemiology based on geographic variation of the virus.

## 2. Case Presentation

A previously healthy, immunocompetent 27-year-old resident of Canada with a history of iron-deficiency anemia presented to the Emergency Department (ED) with a four-day history of intermittent, subjective fevers and a rash. He described the rash as small, slightly raised, red lesions that were intensely pruritic. The rash erupted several hours after the onset of his first fever and was initially localized over his back, but it spread over his arms, chest, stomach, and upper thighs over the next several days. It spared his mouth and genitals. He continued to experience intermittent fevers throughout all four days but did not endorse any other infectious symptoms such as vomiting, diarrhea, pharyngitis, coryza, or drenching night sweats. 

To better characterize his epidemiologic risk, a thorough social history was taken: he was born in Angola and lived there as a child before moving to the DRC, and finally to Canada at age 10, where he settled in the catchment area of our institution. He reported no relevant occupational exposures and had not had any sick contacts in his personal life. He was in a monogamous relationship with a female partner and did not endorse any symptoms or signs concerning for sexually transmitted infection. His partner was well. He reported traveling to the DRC every 2 years on average and his last travel to the DRC was approximately 9 months prior to presentation, where he had stayed visiting friends and family for two months. He was infected with malaria during his stay and treated for it while there. Beyond the earlier trip back to the DRC, his last travel outside of Canada had been to Belgium five months earlier when he had traveled for one week for business purposes. He did not contract any illnesses while there and had been in good health prior to and following his trip. Upon presentation to the ED, he was hemodynamically stable and afebrile. At that time, his rash was described as scattered, pearly vesicles with no central umbilication. He was lymphopenic (WBC 2.4 × 10^9^/L; lymphocytes 0.6 × 10^9^/L) and thrombocytopenic (platelets 135 × 10^9^/L). Given his presentation and epidemiology, he underwent testing for malaria via a rapid diagnostic test (RDT) and thick and thin blood film examination. He was tested for respiratory viruses (including influenza A and B, RSV, and SARS-CoV-2) via nasopharyngeal swab, Mpox, HSV-1/2, and VZV via a PCR of swabbed vesicular exudate (per the differential diagnosis outlined in Table 1), and *Chlamydia* and gonorrhea testing of urine. He was then referred to our outpatient unit for further work-up and management. The results of his blood work and infectious investigations drawn in the ED are included in Table 2. 

**Figure 1:**
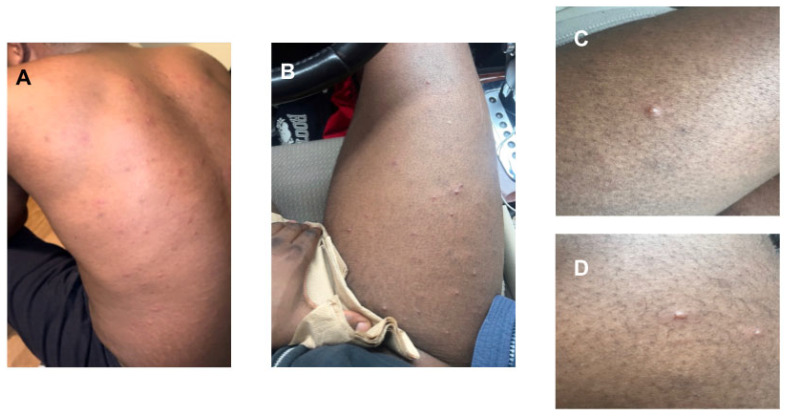
Umbilicated and vesicular rash on Days 4–5 of symptoms. (**A**)—Backside of patient on Day 4, during his emergency department visit. (**B**)—Right thigh of patient on Day 5, one day prior to consultation with our unit. (**C**)—Left arm of patient on Day 5 of illness. (**D**)—Close up of right thigh on Day 5 of illness.

**Figure 2:**
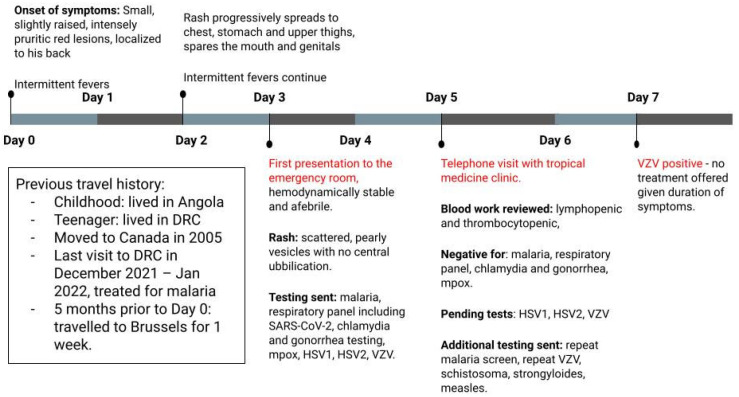
Timeline depicting the onset and progression of symptoms and events leading to diagnosis and resolution.

Two days following his ED visit, the patient was assessed in our unit. At that time, the frequency of his fevers had decreased, and he noted the evolution of his lesions with many of them being crusted over and scabbed (Figure 1). Malaria screen, respiratory virus detection panel, *Chlamydia* and gonorrhea urine testing, and Mpox viral PCR were all negative at the time of consultation in our unit, and his HSV-1/2 and VZV PCR testing was pending. Further investigations were added to his work-up, including a repeat malaria screen, measles serologies, as well as serologic screening for *Schistosoma* and *Strongyloides* based on place of birth and childhood residence. A clinical diagnosis of probable primary Chickenpox was rendered, and the patient was counseled to rest, remain hydrated, remain off work, and use over-the-counter analgesics as needed. As he was already 6 days into the illness at that time, afebrile on examination, clinically improving, and with lesions already crusting over, he was not offered antivirals. He was advised to remain isolated from individuals who may be at risk of more severe sequelae, such as infants, pregnant women, and those who were immunocompromised. Close follow-up was arranged. Figure 2 provides a summary of his disease course. The patient provided informed consent for publication of this case.

On approximately Day 7–8 of symptoms and following full crusting of the lesions, his VZV PCR of an unroofed lesion returned positive, confirming the diagnosis of primary varicella. Measles serology indicated a positive IgG and negative IgM. He recovered fully and without any complication from his varicella infection. 

## 3. Discussion and Mini-Review

We herein report a case of primary varicella infection in an immunocompetent, adult patient originally from Angola and the DRC whose epidemiological risk factors, including his age and countries of birth and residence, prompted a necessary consideration of a broader differential. The diagnosis of pVZV can be made clinically based on the characteristic history and rash, or microbiologically via PCR testing of fluid swabbed from vesicular lesions (Table 1) [9]. In Ontario, outpatient testing is often performed at the Public Health Ontario Laboratory (PHOL), where the turnaround time for VZV PCR is up to 4 days from the sample being received [10]. Once diagnosed, treatment is recommended in immunocompetent adults to prevent complications; though timely treatment is essential with studies demonstrating a lack of effect on outcomes when initiated over 72 h from symptom onset. Unlike pVZV infection in childhood, which is usually mild and self-limiting, infection in adults carries an increased risk of severe complications including pneumonia, bacteremia from secondary skin and soft tissue infections, hepatitis, and encephalitis [6]. Accordingly, case fatality rates are highest amongst adult patients (varicella pneumonia carries a mortality rate of 10–30%, for example) [11]. Acyclovir and its analogues (valacyclovir and famciclovir) are the treatment of choice for primary varicella, with valacyclovir being favored for its reduced pill burden of three times daily dosing compared to acyclovir, which requires five times daily dosing (Table 1). Studies have shown that the benefit of antiviral therapy is greatest when treatment is initiated within 24 h of symptom onset, and that by 72 h following the onset of the rash, most viral replication has stopped in immunocompetent adults [7,8]. As our patient presented to the ED initially at 96 h following the onset of symptoms, and based on rapid clinical improvement, he was not offered antiviral therapy. The early window of antiviral efficacy underscores the importance of making a timely diagnosis of pVZV and initiating therapy early in the course of the infection for the prevention of severe complications.

**Table 1:** Characteristics of viruses manifesting in a similar manner to the varicella zoster virus.

	VZV	Mpox	HSV-1/2	Measles	Coxsackie Virus
Exposure	HHT: contact with active lesions or airborne [12]	1. HHT: contact with mucous membranes or broken skin 2. AHT [13]	HHT: contact with herpetic lesions or mucosal secretions [14]	HHT: contact or airborne transmission [15]	HHT: fecal–oral or contact with oral and respiratory secretions [16]
Incubation period (days)	10–21 days [12]	7–10 days [17]	4–21 days (5–13 commonly) [18]	10–14 up to 23 days [15]	3–6 days [19]
Clinical Manifestations	maculopapular, vesicular rash, and acute neuritis [20] Complications: skin and soft tissue infections, pneumonia, encephalitis, and Reye syndrome [20]	Prodromal symptoms: fever, headache, sore throat, back pain, and myalgia followed by rash (papules, vesicles, or pseudo-pustules frequently present with lesions located on the anogenital and perioral areas) [17] Rare symptoms: Proctitis/tonsillitis and ocular infections [17]	Oral infection with painful oral lesions (gingivostomatitis and pharyngitis) with local lymphadenopathy, genital infections (bilateral genital ulcerations and tender lymphadenopathy, cutaneous manifestations (herpetic whitlow, eczema herpeticum, and herpes gladiatorum) [21]	Prodromal symptoms: fever, malaise, and anorexia, followed by conjunctivitis, coryza, and cough. Followed by enanthem (Koplik spots) and exanthem (erythematous, maculopapular, and a blanching rash, which classically begins on the face) [15]	Mouth or throat pain. Oral enanthem-vesicles surrounded by a thin halo of erythema on the tongue and buccal mucosa [16]
Demographics	Temperate climates: Children (ages 2–8 years) LMIC: adolescents and adults Race: White Americans, with lifetime incidence being lower in Black Americans [22]	Men who have sex with men or identify as transgender/gender diverse [23] Endemic: Cameroon, the Central African Republic, the Democratic Republic of the Congo, Gabon, Ghana (identified in animals only), Cote d’Ivoire, Liberia, Nigeria, the Republic of the Congo, and Sierra Leone [19]	Close (but not necessarily sexual) contact: Family members, and children in day care centers where saliva sharing behavior can occur [14]	Absence of vaccination: children too young, those only with one dose of the measles vaccine, and those who failed to elicit a protective immune response [15]	Young children and infants (higher risk for infection during the first year of life) [19]
Diagnosis	Immunocompetent individuals: clinical presentation. Atypical clinical presentation: Lab confirmation using PCR testing, DFA testing, and viral culture [24]	Serologic testing, viral PCR for *Orthopoxvirus* DNA [25]	PCR testing or viral culture [14]	Serology test for serum measles IgM antibody, significant rise in measles IgG antibody between acute and convalescent titers, Viral Culture, or RT-PCR test of throat and/or urine [26]	Diagnosed clinically based upon the typical appearance and location of the oral enanthem and exanthem [19]
Treatment	Supportive therapy. AVs (acyclovir) can reduce duration and severity of symptoms [12]	Supportive therapy. In individuals with severe disease (involvement of oral mucosa or eyes) AV therapy (tecovirimat) is warranted. Post-exposure vaccination can be warranted [25]	AV agents for HSV infection include acyclovir, valacyclovir, and famciclovir within 72 hrs of symptom onset [14]	Supportive therapy includes antipyretics, fluids, and treatment of bacterial superinfections [26]. Vitamin A for hospitalized inpatients with moderate to severe illness.	Supportive therapy [16]
Prevention	Infection control (isolation, airborne precautions, and hygiene) and vaccination [12]	Pre-exposure prophylaxis (PrEP) with the live, nonreplicating, modified vaccinia Ankara (MVA) vaccine [25]	Patient education, use of barrier protection, and chronic suppressive therapy [14]	Infection control, measles, mumps, and rubella vaccination [26]	Infection control (isolation and hygiene) [18]

Caption: AHT: animal-to-human transmission; AV: antiviral; DFA: direct fluorescent antibody; HHT: human-to-human transmission; HSV, herpes simplex virus; LMIC: low- and middle-income countries; and PCR: polymerase chain reaction.

**Table 2:** Notable blood work on Days 4 and 6 following symptom onset.

Laboratory Parameter	Value	Normal Range
Hemoglobin, g/L	125	140–180
White blood cells, bil/L	2.4	4.0–11.0
Neutrophils, bil/L	1.5	2.0–7.5
Lymphocytes, bil/L	0.6	1.5–4.0
Eosinophils, bil/L	0	<0.4
Platelets, bil/L	135	150–400
Sodium, mEq/L	144	135–145
Chloride, mEq/L	110	96–106
Glucose, mmol/L	5.4	3.9–5.6
Anion gap, mEq/L	8	4–12
Creatinine, umol/L	83	45–110
AST, U/L	24	<33
ALT, U/L	23	<40
Alkaline phosphatase, U/L	68	<110
Bilirubin, umol/L	10	<20 umol/L
**Microbiology**		
Drawn on:	Day 4 of Symptoms	Day 6 of Symptoms
HIV rapid test	Negative	
*Chlamydia* and gonorrhea	Negative	
Malaria screen	Negative	Negative
COVID-19	Negative	
RSV	Negative	
Influenza A and B	Negative	
Mpox	Negative	
HSV PCR		Negative
VZV PCR		Positive

VZV occurs worldwide with a year-round transmission; however, research regarding its epidemiology and disease burden has primarily been studied in high-income countries (HIC) [1]. In Canada, prior to the implementation of its publicly funded childhood vaccination program in 2004, 50% of children by the age of 5 developed pVZV and 90% by the age of 12 years [11]. Since then, annual cases have fallen from approximately 350,000 to less than 1000 [27]. A 2000 Canadian seroprevalence study demonstrated that 89.6% of adolescents aged 15–19, 93.2% of adults aged 25–29, and 96.5% of adults aged older than 40 years had detectable VZV antibodies [28]. A similar epidemiology is reflected in US-based surveillance, which has shown pVZV incidence as being highest in children aged 5–9 years and seroprevalence rates of 66% in children aged 4–5 years [29], while a separate study showed seroprevalence rates of 98.0% in adults aged 20–49 years [30]. Conversely, in tropical climates—where many LMIC are situated—studies have demonstrated a higher mean age of acquisition and lower rates of seroprevalence amongst adults [1]. For example, in Sri Lanka the mean age of acquisition of pVZV was 14.5 years, and a study amongst adult military recruits in Puerto Rico found that 42% of participants were seronegative for VZV [31,32]. Our patient migrated to Canada from the DRC having been born in Angola. In sub-Saharan Africa, VZV continues to be understudied. A recent systematic review of VZV morbidity and mortality from 1974 to 2015 included 20 studies representing only 13 countries [33]. A seroprevalence study performed in the DRC demonstrated seropositivity in only 8% of children aged 6–59 months [34]; meanwhile, another study performed in the Tshuapa province in the DRC demonstrated a mean age of acquisition of 13 years with seropositivity rates of only 34% in adults above the age of 20 [35].

While the differences in varicella epidemiology, including the age of acquisition, between temperate and tropical climates are fairly well described, our understanding of the factors driving these differences remains poorly understood with viral, host, and geo-socio-climatic factors all having likely influence [32]. Different proposals have been made in order to distinguish VZV in separate viral clades based on genotype and geographic origin. Breuer and colleagues describe five viral clades: clades 1, 3, and 5 are of European origin, clade 2 includes strains of Asian origin, and clade 4 contains African strains [23]. Similarly, Barrett-Muir and colleagues in 2003 classified VZV into four genotypes: A (Africa/Asia), B and C (Europe and America), and J (Far East) [36]. Studies have demonstrated that the dominant genotypes circulating in most temperate high- and middle-income countries are clades B and C, which might underpin differences in viral or host factors that account for higher seroprevalence rates amongst adults in these countries [1]. Meanwhile, previous studies have demonstrated that age, household exposure to varicella, number of siblings, school attendance, and area of residence (urban versus rural) are all geo-social factors that have been associated with varicella seropositivity. For example, studies performed in Sri Lanka and India demonstrated that there was a higher level of anti-VZV IgG antibodies amongst adolescents living in an urban area compared with their rural counterparts, possibly reflecting high population density leading to increased rates of transmission [37].

Climatic factors have also been examined with regards to their effects on VZV transmission. It has been proposed that high humidity and ambient temperatures are inversely correlated with varicella incidence, which has been supported by various studies performed in countries with both temperate and tropical climates [38]. For example, a study performed in Jinan, China, demonstrated a weekly decrease in varicella incidence by 3.4% for every 1 °C increase in ambient temperature [29]. A separate hypothesis has been proposed suggesting that ultra–violet (UV) radiation inactivates VZV within vesicles on exposed skin prior to or following rupture, thus reducing infectivity [32,39,40]. These hypotheses together might explain why transmissibility in LMICs with tropical climates remains low throughout the year and peaks in HICs with temperate climates in winter and early spring [1,32].

Our case similarly highlights the overlapping clinical presentations between primary varicella infection and other common and emerging viral infections, including Mpox (Table 1). Notably, viral exanthems can be difficult to distinguish from one another and often clinical and epidemiologic clues are required to promptly make a diagnosis while awaiting laboratory testing. Mpox is an *Orthopoxvirus* endemic to West and Central Africa that was responsible for an international outbreak in 2022–2023 [4]. Its clinical presentation and characteristic rash most commonly resemble that of smallpox, another *Orthopoxvirus* that has been eradicated globally; the appearance of Mpox can be mistaken for primary varicella [4]. There are several differences, however, that help distinguish Mpox from primary VZV infection (Table 1) [4]. These include a fever that may occur 1–3 days prior to rash onset with lymphadenopathy in Mpox versus fever 1–2 days prior to rash onset and the absence of lymphadenopathy in primary varicella [29]. The rashes themselves characteristically appear differently with Mpox lesions being umbilicated with regular, defined borders, whereas varicella lesions are often superficial with irregular borders. In varicella, the lesions are often present at multiple stages (as was the case in our patient), whereas in Mpox, the lesions generally evolve together and so all lesions will be in the same stage of infection [4,40]. VZV and Mpox are both endemic to DRC, from where our patient had migrated, and several studies looking at Mpox incidence in Central Africa have identified VZV without PCR evidence of Mpox where a clinical diagnosis of Mpox had been made [35]. Similarly, a recent study from 2021 performed in the Tshuapa province identified 9.4% of patients being co-infected with both Mpox and VZV at the same time [4,38]. Patients with co-infection frequently reported rash, fever, fatigue, chills, headache and myalgias. They were significantly more likely to report fatigue, conjunctivitis, and being “bed-ridden” compared with patients with VZV mono-infection; conversely, they were less likely to report mouth sores, axillary lymphadenopathy, cough, sore throat, and being “bedridden” when compared with patients with Mpox mono-infection [38]. The authors also compared rash characteristics between groups and found that patients with co-infection were significantly less likely to have lesions on their face, thorax, arms, palms, and soles than the Mpox group, and more likely to have a higher rash burden than the VZV mono-infection group [29]. This further highlights the importance of understanding the differences in epidemiology and clinical presentation for primary varicella and other common viral exanthems in order to ensure a sufficiently broad differential diagnosis, adequate specimen collection and testing, and a prompt initiation of appropriate therapy, if clinically indicated.

In summary, though commonly considered a disease of childhood, primary VZV has a diverse epidemiology and can mimic other common viral infections, challenging the certainty of clinical diagnoses. Key clinical characteristics are important for distinguishing pVZV from other common viruses and diffuse cutaneous eruptions (Table 1). Meanwhile, it is important to recognize the differences in VZV epidemiology, such as the age of presentation and population-wide seroprevalence between countries with a temperate climate versus a tropical/sub-tropical climate, which can change the pre-test probability and the decision to test for various viruses in returning travelers manifesting a dermatosis. This especially holds true in the context of the global Mpox outbreak, the clinical presentation of which can mimic primary varicella and whose endemicity overlaps with VZV’s global distribution.

## 4. Conclusions

The case reported herein illustrates the challenge of diagnosing primary varicella infection in adults, as well as differences in epidemiology based on geographic variation of the virus. The case further highlights the need for microbiological testing in adults to confirm VZV infection in the Mpox era. Although our patient had a mild clinical course of VZV, adults are at risk of complications and may require treatment with anti-virals to mitigate such risks.

## Data Availability

All data are presented in the manuscript.
